# Impact of delays in initiating postoperative chemoradiation while determining the MGMT promoter-methylation statuses of patients with primary glioblastoma

**DOI:** 10.1186/s12885-015-1545-x

**Published:** 2015-07-30

**Authors:** Sebastian Adeberg, Tilman Bostel, Semi Harrabi, Denise Bernhardt, Thomas Welzel, Wolfgang Wick, Juergen Debus, Stephanie E. Combs

**Affiliations:** 1 Department of Radiation Oncology, University Hospital of Heidelberg, Im Neuenheimer Feld 400, 69120 Heidelberg, Germany; 2 Heidelberg Ion-Beam Therapy Center (HIT), Im Neuenheimer Feld 450, 69120 Heidelberg, Germany; 3 Clinical Cooperation Unit Radiation Oncology, German Cancer Research Center (DKFZ), Im Neuenheimer Feld 280, 69120 Heidelberg, Germany; 4 Department of Neurooncology, University Hospital of Heidelberg, Im Neuenheimer Feld 400, 69120, Heidelberg, Germany; 5 Department of Radiation Oncology, Klinikum rechts der Isar, Technische Universität München, Ismaninger Str. 22, 81675, München, Germany

**Keywords:** MGMT promoter, Glioblastoma, Treatment delay, Radiation therapy

## Abstract

**Background:**

The benefits of new innovations in glioblastoma therapies should not be curtailed as a result of delays in commencement of radiation therapy, caused by clinical circumstances as well as diagnostic procedures. This study evaluates whether delays in chemo-radiotherapy after surgery, while determining O6-methylguanine-DNA-methyltransferase (MGMT) promoter status, affect the survival rates of patients with glioblastoma (GBM).

**Methods:**

Our sample comprised 50 GBM patients in a retrospective analysis of three prospective studies that focused on combined radiotherapy and required MGMT promoter-status testing as inclusion criteria. Results were compared with a reference group of 127 favourable GBM cases (Karnofsky performance-status scale ≥ 70), in which the patients underwent standard postoperative chemo-radiotherapy with temozolomide. Survival time was calculated using the Kaplan-Meier method, and a multivariate analysis of the delays between surgical and radiotherapy procedures was performed using the Cox regression model.

**Results:**

The study group’s median overall survival time was 16.2 months (with a range of 2 to 56 months), versus the reference group’s survival time of 18.2 months (with a range of 1 to 92 months) (*p* = 0.64). The delay between surgery and radiotherapy was increased by 8 days in the study patients (p < 0.001), with a median delay of 35 days (range: 18–49 days) corresponding to the typical 27-day delay (range: 5–98 days) for those in the reference group. Univariate and multivariate analyses did not show any negative association between survival time and delaying radiation therapy to determine MGMT-promoter status; commencement of radiation therapy sooner than 24 days after surgery was the threshold for significantly decreased overall survival (*p* = 0.01) and progression-free (*p* = 0.03) survival.

**Conclusion:**

Delaying postoperative chemoradiation for GBM patients—carried out in order to determine MGMT-promoter status—did not have a negative impact on survival time. Indeed, the data of the present study shows that initiating radiation therapy sooner than 24 days after surgery has a negative impact on progression and survival.

## Background

GBM is the most common type of primary brain tumours in adults. Standard therapy for this type of cancer consists of maximum surgical resection, followed by postoperative temozolomide-based (TMZ-based) chemoradiation [1]. And yet, survival time—with a median of around 15 months—is still unsatisfactory for this therapy regimen [1].

Despite the discovery of new therapeutic approaches, delayed initiation of treatment has been shown to have a negative impact on survival rates; this hypothesis has recently garnered renewed attention, as data on other tumour types (e.g. breast cancer [2–4], head and neck cancer [4, 5], and small-cell lung cancer [6, 7]) have supported it. The mechanisms underlying this phenomenon are still not entirely understood. The physical effects of treatment delay—including early, local recurrence and distant relapse—might be described as increasing in probability as a function of time [8]. It seems obvious that effects might be more pronounced in fast-growing tumours with short doubling times [8].

Because GBMs are highly aggressive, quickly proliferating tumours, the connection between delay in radiotherapy and decreased survival for GBM patients has led to the general practice of initiating treatment about 2 to 4 weeks after surgery. One can expect further progression to be severely affected by a delay in treatment initiation [8, 9]. Previous studies implicate delayed radiotherapy in a significant reduction of survival times for high-grade glioma patients [10, 11].

Hence, GBM progression—with its aggressive characteristics—may be highly receptive to the negative effects caused by delays in initiation of treatment. And yet, there are many potential reasons for delaying treatment, including surgical complications, limited resources, and comorbidities.

Longer delays in initiation of treatment can also be linked to elaborate, highly advanced treatment regimens (e.g. time is needed to determine molecular markers). These markers and their activation statuses have been known to stratify into different treatment groups. For GBM, MGMT-promoter methylation is known to influence survival after chemoradiation with TMZ. That is why several studies based on this stratification factor have already been performed [12].

However, determination of the MGMT-promoter methylation status is difficult [13] and time-consuming. Several European Organisation for Research and Treatment of Cancer (EORTC) studies have recently been conducted to evaluate new therapeutic approaches connected to MGMT methylation status [14].

The present study evaluates a large number of GBM patients treated with TMZ chemoradiation and analyses how differing times between their surgeries and treatments affected their outcomes. The aim of this analysis is to show whether increasing the span of time between surgery and chemoradiation in order to determine MGMT-promotor methylation status has a potentially detrimental effect on outcomes.

## Methods

### Study-population selection

Subjects for this study’s analysis were identified using an in-house radiation-oncology brain-tumour database. The study group consisted of 50 patients treated with chemoradiation for histologically proven GBM in multicentre clinical trials (Table 1) at Heidelberg University Hospital’s Department of Radiation Oncology. The main eligibility criteria for this study included those patients who have undergone chemoradiation. Thus, the data are based on a well-characterized and homogeneous population. Next, the MGMT-promoter methylations of the 50 patients in the present study’s sample were compared with those of 223 GBM patients whom the aforementioned Radiation Oncology Department treated primarily with chemoradiotherapy between January 2004 and December 2011. To account for its more selective sampling, the present study only focused on a favourable patient reference group (*n* = 127), with a Karnofsky performance-status scale (KPS) ≥70.

**Table 1:** Study protocols and included patient numbers

Designs of the analyzed prospective studies				
Study	Phase	Treatment regimens	MGMT promoter status	Absolut patient numbers
TEMSIROLIMUS (EORTC 26082–22081)	III	1. Conventional RT with 60 Gy plus temsirolimus 25 mg once a week beginning 7 days before initiation of radiotherapy. Adjuvant cycles of temsirolimus 25 mg once a week until progression.	Unmethylated	19
		2. Conventional RT with 60 Gy plus 75 mg/m2/d TMZ during RT. Up to six cycles of adjuvant TMZ for 5 days every 28 days with 150 mg/m2/d for the first adjuvant cycle and 200 mg/m2/d for the following cycles.		
ENZASTAURIN (H6Q-MC-S039)	II	1. Conventional RT with 60 Gy plus Enzastaurin 250 mg b.i.d. until progression or a maximum of 3 years.	Unmethylated	13
		2. Conventional RT with 60 Gy plus 75 mg/m2/d TMZ during RT. Up to six cycles of adjuvant TMZ for 5 days every 28 days with 150 mg/m2/d for the first adjuvant cycle and 200 mg/m2 for the following cycles.		
CENTRIC (EORTC 26071–22072)	III	1. Conventional RT with 60 Gy plus 75 mg/m2/d TMZ during RT plus Cilengitide 2000 mg twice a week beginning 7 days before initiation of radiotherapy until day 77 or progression. Up to six cycles of adjuvant TMZ for 5 days every 28 days with 150 mg/m2/d for the first adjuvant cycle and 200 mg/m2/d for the following cycles.	Methylated	18
		2. Conventional RT with 60 Gy plus 75 mg/m2/d TMZ during RT. Up to six cycles of adjuvant TMZ for 5 days every 28 days with 150 mg/m2/d for the first adjuvant cycle and 200 mg/m2/d for the following cycles.		

*Abbreviations*: *MGMT* = O6-methylguanine-DNA methyltransferase; *TMZ* = Temozolomide; *mg* = Milligram; *m* = Meter; *d* = Day, *RT* = Radiation Therapy

MGMT-promoter methylation status was assessed using a methylation-specific (MSP) chain reaction, as previously described [15]. KPS was determined before initiating radiation therapy.

### Study-group treatment regimens

Result summaries for the three clinical trials used in the present study are depicted in Table 1. Prior to randomization and result measurements, written, informed consent was obtained from each participating patient. In all three studies, conventional, 3D-conformal fractionated radiotherapy was performed, and the protocols of all three studies, as well as the current retrospective study (Nr. S-056/2015) were approved by Heidelberg University Hospital’s ethics committee. All studies were performed in accordance with local laws and the Helsinki Declaration and written informed consent was obtained for all study patients.

### Chemoradiotherapy for the reference group

Conventional, 3D-conformal fractionated radiotherapy for the reference group was performed as previously described, with a median dose of 60.0 Gy [16]. For purposes of planning treatment, patients were fixed with custom-made masks, and computed tomography and MRI imaging were performed. Several fractionation schemes were used during the investigation period. Three dimensional-conformal radiation therapy was used on all patients included in the current study. Additionally, patients received concomitant TMZ administration in accordance with the Stupp scheme [1].

### Imaging procedures

Initial MRIs, treatment-planning CTs and MRIs, and follow-up MRIs served as disease-evaluation tests for the entire sample. Tumour localization and progression were determined on the basis of contrast-enhanced, T1-weighted sequences on axial and coronal images. Images were adjudged and reviewed by experienced radiological specialists (T. W. and T. B.).

Clinical hospital course records and pre- and post-operative MRI imaging were evaluated in accordance with institutional guidelines. The time interval between surgery and radiotherapy was calculated in days from the last day of surgical treatment to the first day of radiotherapy. Survival was measured from the date of initial surgical resection to disease progression and last follow-up or death.

Progression-free survival (PFS) was calculated as the timespan between the first day of radiation therapy and the occurrence of local or distant progression, determinations of which were based on contrast-enhanced, T1-weighted MRI scans (axial and coronal) as per RANO criteria. If the radiologist suspected pseudo-progression, further follow-up MRIs were obtained to verify true radiographic progression [17]. Progression analysis data were censored when death occurred without MRI-determined progression, or without a follow-up examination.

Survival analyses were performed using the Kaplan-Meier method. A comparison between the two subgroups was undertaken using the log-rank analysis method. Variable patient characteristics were compared using odds ratios, the *χ*^2^ test, the Cox regression model, and corresponding 95 % confidence intervals. The threshold significance level was *p* < 0.05.

## Results

Patient characteristics are listed in Table 2. The reference group showed an insignificantly higher ratio of patients (OR = 1.5; 95 % CI: 0.77–2.93; *p* = 0.32) who underwent gross-total surgical resection (*n* = 58; 45.7 %), in contrast with the study group (*n* = 18; 36.0 %). Biopsies for pathological confirmation with no further surgery were performed on nine patients (7.1 %) in the reference group and two patients (4.0 %) in the study group. No difference was observed in the composition of both treatment groups in terms of pre-therapeutic, multifocal GBM occurrence (OR = 0.70; 95 % CI: 0.28–1.91; *p* = 0.63).

**Table 2:** Patient characteristics of the study group and reference group

	Study group in absolut numbers (%)	Reference group in absolut numbers (%)
Gender		
Female	16 (32)	45 (35.4)
Male	34 (68)	82 (64.6)
Age in years [range]	58,9 [30.3–75.9]	58.7 [20.3–75.5]
Median KPS [range]	90 [60–100]	90 [70–100]
MGMT-promoter-status		
MGMT-status not determined	0 (0)	95 (74.8)
MGMT-status determined	50 (100)	32 (25.2)
MGMT-promoter methylated	18 (36)	14 (43.8)
MGMT-promoter not methylated	32 (64)	18 (56.2)
Resection status		
Gross Total resection	18 (36)	58 (45.7)
Subtotal resection	30 (60)	60 (47.2)
Biopsy	2 (4)	9 (7.1)
Temozolomide therapy	26 (52)	127 (100)
Study medication	33 (66)	n.e.
Median delay to RT in days [range]	35 [18–49]	27 [5–98]

*Abbreviations: MGMT*: O^6^-methylguanine-DNA-methyltransferase, *KPS*: Karnofsky performance status; *RT*: Radiation therapy

Numbers in parentheses (*) are the percentages

The median survival time for all patients in the study was 16.2 months (with a range of 2 to 56 months), versus a survival time of 18.2 months (with a range from 1 to 92 months) for participants in the reference group (*p* = 0.64). There was no significant, verifiable difference in both groups’ progression-free survival (PFS) (6.9 vs. 6.3 months; *p* = 0.20).

In analysing the impact of surgical-resection status on outcome—bearing in mind the higher proportion of patients in the reference group who underwent gross-total resection—the present study compared patients in the reference group after complete (*n* = 58; 45.7 %) or partial resection (*n* = 60; 47.2 %) with patients in the study group (*n* = 18; 36.0 % and *n* = 30; 60.0 %; OR: 1.61, *p* = 0.17) with regard to overall survival (OS) and PFS. No difference in survival rates was found between subtotal-resected patients in the study group (OS: 17.0 months; range: 2.1–55.6 months) and the reference group (OS: 15.4 months; range: 2.7–87.7 months) with *p* = 0.47. Accordingly, there was no significant difference (*p* = 0.84) in the survival rates of total-resected patients in the study group (OS: 15.8 months; range: 7.3–47.0 months) and the reference group (OS: 22.3 months; range: 2.5–91.7). Study medication did not affect PFS (*p* = 0.59) or OS (*p* = 0.88) in the study group.

Interestingly, the results of the present study showed a statistically significant difference in outcomes for the two groups, based on the differing time intervals between surgery and the start of radiotherapy (p < 0.001). The median delay between these two treatments for study patients was 35 days (with a range of 18 to 49 days), versus the reference-sample patients’ median delay of 27 days (range: 5–98 days). The median additional delay for MGMT-promoter determination was 8 days (with a range of 3 to 34 days). MGMT methylation status significantly influenced OS in the reference group (methylated: 25.2 months vs. non-methylated: 15.4 months; *p* = 0.04), whereas this, in no way, influenced survival for the study group (*p* = 0.129). The results of this study’s univariate and multivariate analyses are shown in Tables 3, 4 and 5. Delay was found to have had no significant negative effects on OS and PFS. However, delaying radiotherapy had a significant protective effect on death rates, with a Hazard ratio of 0.95 (95 % CI: 0.90–1.00; *p* = 0.044) (Table 5). Interestingly, this study’s authors observed a threshold for decreased progression-free and overall survival in both groups if radiotherapy was performed earlier than 24 days after surgery.

**Table 3:** Univariate proportional-hazards regression analysis of cofactors on progression-free survival in glioblastoma patients

Cofactors	HR	95 % CI	*P* value
Study group			
Age <60 years	0.73	0.39–1.35	0.31
Karnofsky Performance Status >70	0.65	0.25–1.67	0.37
Resection	0.24	0.03–1.92	0.18
Gross Total Resection	0.91	0.48–1.74	0.77
Study medication	1.19	0.63–2.26	0.59
MGMT promoter methylation	1.23	0.64–2.35	0.54
Reference group			
Age <60 years	1.07	0.70–1.64	0.76
Karnofsky Performance Status >70	n.e.	n.e.	n.e.
Resection	1.27	0.47–3.48	0.64
Gross total Resection	0.85	0.56–1.29	0.45
MGMT promoter methylation	0.35	0.15–0.86	0.02

Abbreviations: *CI* = Confidence interval; *HR* = Hazard ratio; *SVZ* = Subventricularzone; *MGMT* = O6-methylguanine-DNA methyltransferase

**Table 4:** Univariate proportional-hazards regression analysis of cofactors on overall survival in glioblastoma patients

Cofactors	HR	95 % CI	*P* value
Study group			
Age <60 years	1.18	0.62–2.25	0.62
Karnofsky Performance Status >70	1.00	0.35–2.84	0.99
Resection	0.81	0.44–1.54	0.72
Gross Total Resection	1.09	0.56–2.12	0.81
Study medication	0.95	0.48–1.87	0.88
MGMT promoter methylation	0.63	0.31–1.27	0.19
Reference group			
Age <60 years	0.78	0.27–2.61	0.73
Karnofsky Performance Status >70	n.e.	n.e.	n.e.
Resection	0.81	0.35–1.85	0.61
Gross Total Resection	0.75	0.50–1.12	0.16
MGMT promoter methylation	0.43	0.18–0.99	0.048

Abbreviations: *CI* = Confidence interval; *HR* = Hazard ratio; *SVZ* = Subventricular zone; *MGMT* = O6-methylguanine-DNA methyltransferase

**Table 5:** Univariate proportional-hazards regression analysis of the influence of treatment delay on progression-free and overall survival in glioblastoma patients

Group	Survival	Covariate	HR	95 % CI	Significance level
Study group	OS	Time delay to RT	0.95	0.90–0.99	0.04
Reference group	OS	Time delay to RT	0.99	0.98–1.00	0.13
Study group	PFS	Time delay to RT	0.97	0.93–1.01	0.18
Reference group	PFS	Time delay to RT	1.00	0.98–1.01	0.59
Study group	OS	Time delay to RT ≥24 days	0.43	0.23–0.83	0.01
Reference group	OS	Time delay to RT ≥24 days	0.59	0.40–0.89	0.01
Study group	PFS	Time delay to RT ≥24 days	0.49	0.26–0.92	0.03
Reference group	PFS	Time delay to RT ≥24 days	0.62	0.40–0.96	0.03

*OS*: Overall survival; *PFS*: Progression-free survival; *HR*: Hazard ratio; *CI*: Confidence interval; *RT*: Radiation therapy

## Discussion

This study found an 8-day increase in the median time interval between surgery and the initiation of radiotherapy due to MGMT-promoter methylation determination. However, its results showed that delaying radiation therapy to determine MGMT-promoter methylation did not have a significantly negative impact on patients’ survival. Interestingly, study participants actually exhibited a decreased likelihood of death in dependency-of-treatment delay (HR: 0.95; *p* = 0.045). Thus, initiating RT earlier than 24 days after surgery was associated negatively with overall survival.

The association between delaying radiation and decreased survival, as well as higher loco-regional recurrence rates, has been examined most extensively for head-and-neck, breast, and small cell-lung cancers [2–7]. Unfortunately, contradictory findings have been reported for GBM. Time delays between GBM patients’ undergoing surgical procedures and radiotherapy at Heidelberg University Hospital’s Department of Radiation Oncology is usually about 3 to 5 weeks; nevertheless, the ideal time interval is yet to be determined. A study published in 2000 first explicitly evaluated the effects of radiotherapy delays on the progression of high-grade glioma. In a single institutional trial, the authors reported that waiting for radiotherapy had a negative impact on patient survival rates and risk of death increased by 2 % for every day radiation therapy was delayed. This earlier study’s sample comprised 182 GBM patients who were subject to a median delay of 26 days [18], and its findings should be considered with some degree of reservations because of the 2007 revisions to the neuropathological classifications [19] and because it selected its sample-patient population by removing favourable prognostic cases [18]. Indeed, Thomson et al. found no relationship between delaying radiation therapy and treatment outcomes for GBM patients [20]. Likewise, a review of 16 RTOG-study populations, comprising 2855 GBM patients, could not find any evidence of reduced survival rates caused by increased wait times (up to 6 weeks) for radiotherapy.

Still, patients who started radiotherapy more than 4 weeks after surgery showed a significant survival advantage when compared with those who began radiation therapy within 14 days of undergoing surgery. These unexpected findings might be explained by the fact that postoperative radiotherapy is typically initiated more immediately for advanced tumours, thus contributing to the larger number of patients in the sample with KPS ≤ 70. Hence, there was an overrepresentation of study-group patients who only underwent biopsies for GBM confirmation [21]. A similar approach might explain the results of the present study, which showed decreased survival rates for those who underwent radiation therapy sooner than 24 days after surgery: physicians might simply initiate radiation therapy sooner for more pronounced cases of GBM. And yet, median KPS for this subgroup in the present study’s sample was 90. Commencing radiation therapy later than the abovementioned 24 days might, therefore, seem to have served as a protective factor. It seems obvious, though, that those with highly prolific tumours, like GBM, should receive post-operative radiation therapy as soon as possible. Therefore, unnecessarily delaying radiation therapy should be avoided, even though the present study’s results do not state this; previous data also suggest radiation therapy in cases of malignant supratentorial glioma should take place no later than 37 days after surgical resection. Prolonging wait times further than this has led to significant decreases in overall survival rates [22].

Interestingly, Irwin et al. found a statistically significant increase in death rates (8.9 %) for every additional week between surgery and radiotherapy. It should also be noted that around 50 % of patients experienced a treatment delay of 5 weeks or more, due to personal and financial constraints. In fact, disproportionate radiotherapy delays might explain diminished survival rates [10]. To be sure, the data of this study did not show that such delays negatively impacted survival for members of its own sample or those in its reference sample. Yet, the short delays for persons in the sample of the present study (with a median of 5 weeks and a range of 1 to 7 weeks) make it difficult to draw comparisons. It is as yet unclear, at any rate, if the delays to RT caused by histological examinations and new technologies—as were mandatory for enrolment in the utilized studies—negatively impact survival.

Also notable in all three examined studies were the applications of new therapeutic approaches that used cytotoxic and immune-modulating agents. Therapeutic advances should not, therefore, be relativized to clinical circumstances, such as time delays for MGMT-promoter determination, as mandated by this study’s inclusion criteria. The present study’s authors must emphasize the creation of reference groups that suit inclusion criteria. To minimize potential bias by varying patient characteristics KPS ≥ 70, resection status, and concomitant TMZ therapy were selected to create two consistent groups and facilitate comparison. However, no study has been designed to evaluate delays between surgical resection and the initiation of radiotherapy. The majority of study patients received a study medication, whereas the reference group was given TMZ therapy, and this might have caused biased results. Still, the application of study medication was not associated with patient survival. And, although the present data suffers from the potential bias and usual shortcomings inherent in retrospective study designs, prospective trials evaluating the relationship between delays and survival are not practical.

Finally, the premature commencement of radiotherapy after surgical procedures might be counterproductive, and radio-sensitivity is likely to be diminished by hypoxia and oedematous areas in the surgical site [21]. Technical innovations offer new approaches for treating the GBM; paradoxically, recent steps that subtly lengthen the interval between surgery and the commencement of radiation therapy do not seem to endanger therapy success.

## Conclusion

Granting the inherent limitations of retrospective analyses, the present study found that delays between surgery and radiation treatment for GBM patients, as caused by MGMT-promoter determination, were not disadvantageous; rather, starting radiation therapy sooner than 24 days after surgery was an independent negative prognostic survival factor. And yet, these findings only reflect data from patients whose radiation therapy was initiated within 7 weeks after surgery. Logic dictates that delaying treatment beyond a certain point will negatively influence survival, but this was not examined in the present study. Determining the optimal delay between surgery and radiation therapy is, therefore, of great interest, and merits further investigation.
